# Ethyl 4-hydroxy­methyl-2-methyl­pyridine-5-carboxyl­ate

**DOI:** 10.1107/S160053680801026X

**Published:** 2008-04-23

**Authors:** Peter D. W. Boyd, Gersande Lena, Julie A. Spicer

**Affiliations:** a Department of Chemistry, The University of Auckland, Private Bag 92019, Auckland, New Zealand; b Cancer Research Laboratory, The University of Auckland, Private Bag 92019, Auckland, New Zealand

## Abstract

The title compound, C_10_H_13_NO_3_, was obtained as a by-product of the aldolization reaction of furo[3,4-*c*]pyridin-3(1*H*)-one with thio­phene-2-carboxaldehyde. The substituents on the pyridine ring are nearly coplanar, with an 8.1 (2)° rotation of the hydroxmethyl group from this plane. The mol­ecules assemble in the crystal structure as chains *via* O—H⋯N hydrogen bonding between the pyridine N atom and a neighbouring hydroxy­methyl OH group.

## Related literature

For related literature, see: Goswami *et al.* (2006 ▶), Wu *et al.* (2006 ▶). For bond-length data, see: Allen *et al.*, (1987 ▶).
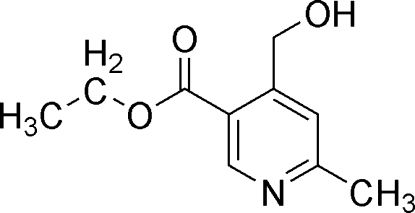


## Experimental

### 

#### Crystal data

C_10_H_13_NO_3_
*M* *_r_* = 195.21
Monoclinic, 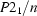

*a* = 4.4998 (2) Å
*b* = 15.4499 (8) Å
*c* = 14.2036 (7) Å
β = 96.417 (1)°
*V* = 981.27 (8) Å^3^
*Z* = 4
Mo *K*α radiation
μ = 0.10 mm^−1^
*T* = 87 (2) K
0.32 × 0.18 × 0.12 mm

#### Data collection

Siemens SMART CCD diffractometer
Absorption correction: none
5759 measured reflections
1987 independent reflections
1786 reflections with *I* > 2σ(*I*)
*R*_int_ = 0.081

#### Refinement

*R*[*F* ^2^ > 2σ(*F* ^2^)] = 0.049
*wR*(*F* ^2^) = 0.134
*S* = 1.02
1987 reflections
130 parameters
H-atom parameters constrained
Δρ_max_ = 0.30 e Å^−3^
Δρ_min_ = −0.28 e Å^−3^

### 

Data collection: *SMART* (Siemens, 1995 ▶); cell refinement: *SAINT* (Siemens, 1995 ▶); data reduction: *SAINT*; program(s) used to solve structure: *SIR92* (Altomare *et al.*, 1993 ▶); program(s) used to refine structure: *SHELXL97* (Sheldrick, 2008 ▶); molecular graphics: *ORTEP-3 for Windows* (Farrugia, 1997 ▶) and *Mercury* (Macrae *et al.*, 2006 ▶); software used to prepare material for publication: *WinGX* (Farrugia, 1999 ▶).

## Supplementary Material

Crystal structure: contains datablocks global, I. DOI: 10.1107/S160053680801026X/bt2697sup1.cif

Structure factors: contains datablocks I. DOI: 10.1107/S160053680801026X/bt2697Isup2.hkl

Additional supplementary materials:  crystallographic information; 3D view; checkCIF report

## Figures and Tables

**Table 1:** Hydrogen-bond geometry (Å, °)

*D*—H⋯*A*	*D*—H	H⋯*A*	*D*⋯*A*	*D*—H⋯*A*
O3—H3⋯N1^i^	0.82	2.01	2.8227 (17)	170

Symmetry code: (i) 
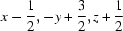
.

**Fig. 1.**
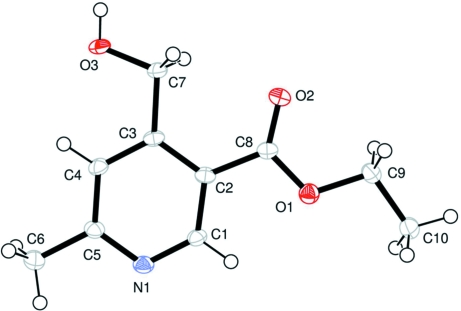
Structure of (I) showing 50% probability displacement ellipsoids for non-hydrogen atoms and hydrogen atoms as arbitary spheres.

**Fig. 2.**
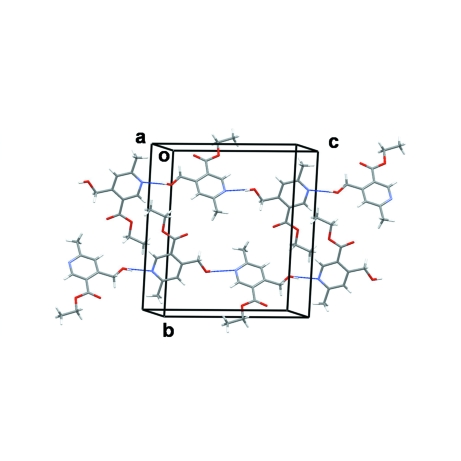
Illustration of the arrangement of the complex (I) in the crystal along the a axis showing pyridine N···H—O hydrogen bonding arrangement.

**Fig. 3.**
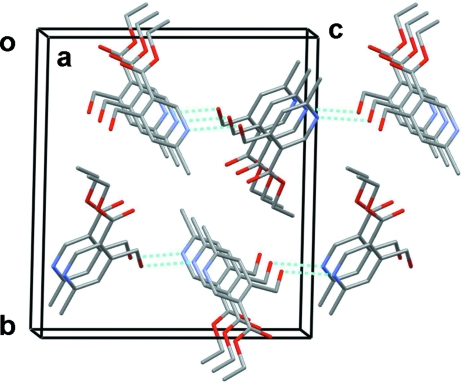
Illustration of the arrangement of the complex (I) in the crystal along the a axis showing stacking of hydrogen bonded chains.

## supplementary crystallographic information

### Comment

The molecular structure of the title compound is shown in Fig. 1. The bond lengths and angles are normal (Allen *et al.*, 1987). The ethyl ester group is nearly coplanar with the pyridine ring (C1-C5,N1 rmsd 0.0064 Å; C2,C8,C9,C10,O1,O2 rmsd 0.0064 Å, interplanar angle 2.17 (9)°). The hydroxymethyl group is rotated slightly out of the plane (O3—C7—C3—C4 8.1 (2)°).

The molecules  in the crystal are connected *via* hydrogen bonding between the pyridine N atom and an adjacent OH group (Table 1) to give chains along the *c* axis (Figure 2a). These chains are stacked along the *a* axis (Figure 2 b). Similar hydrogen bonding interactions are observed in other hydroxymethyl substituted pyridines (Goswami *et al.*, 2006, Wu *et al.*, 2006).

### Experimental

The title compound  was obtained as a by-product of the aldolization reaction of furo[3,4-*c*]pyridin-3(1*H*)-one with thiophene-2-carboxaldehyde. The desired product was not isolated, only the starting material and the title compound   were characterized after the reaction.

Ethyl 4-(hydroxymethyl)-6-methylnicotinate (I): Furo[3,4-*c*]pyridin-3(1*H*)-one (II) (110 mg,0.74 mmol, 1 eq.) was suspended in EtOH (15 ml) at 65°C. Thiophene-2-carboxaldehyde (III) (99 mg, 0.88 mmol) and triethylamine (18 mg,0.18 mmol) were then added and the reaction mixture stirred at 80°C for 6 days. After cooling to room temperature the reaction was quenched with 1*M* HCl and extracted with EtOAc. The organic layer was rinsed with water and dried over MgSO4. Removal of MgSO_4_ by filtration and evaporation of solvent under reduced pressure gave the crude product. This product was dissolved in dichloromethane and stored at 4°C to yield colorless crystals (25 mg, 17% yield) which were isolated by filtration and identified as the title compound. ^1^H NMR (400 MHz, CD_3_)_2_SO, 298 K) δ 8.83 (s, 1 H), 7.03 (s, 1 H), 5.43 (s, 1 H), 4.83 (br s, 2 H), 4.30 (q, J = 7.1 Hz, 2 H), 2.54 (s, 3 H), 1.32 (t, J = 7.1 Hz, 3 H). LCMS (APCI^+^) calcd for C_10_H_13_NO_3_ 195 (MH^+^), found 196.

### Refinement

Hydrogen atoms were placed in calculated positions and refined using the riding model [O—H 0.82 Å, C—H 0.93–0.97 Å), with *U*_iso_(H) =  1.5 times *U*_eq_(O) and *U*_iso_(H) = 1.2 or 1.5 times *U*_eq_(C).

### Crystal data

**Table:**

C_10_H_13_NO_3_	*F*(000) = 416
*M**_r_* = 195.21	*D*_x_ = 1.321 Mg m^−^^3^
Monoclinic, *P*2_1_/*n*	Mo *K*α radiation, λ = 0.71073 Å
*a* = 4.4998 (2) Å	Cell parameters from 4149 reflections
*b* = 15.4499 (8) Å	θ = 2.0–26.3°
*c* = 14.2036 (7) Å	µ = 0.10 mm^−^^1^
β = 96.417 (1)°	*T* = 87 K
*V* = 981.27 (8) Å^3^	Needle, colourless
*Z* = 4	0.32 × 0.18 × 0.12 mm

### Data collection

**Table:**

Siemens SMART CCD diffractometer	1786 reflections with *I* > 2σ(*I*)
Radiation source: fine-focus sealed tube	*R*_int_ = 0.081
graphite	θ_max_ = 26.3°, θ_min_ = 2.0°
Area–detector ω scans	*h* = −5→5
5759 measured reflections	*k* = −19→17
1987 independent reflections	*l* = −17→12

### Refinement

**Table:**

Refinement on *F*^2^	Primary atom site location: structure-invariant direct methods
Least-squares matrix: full	Secondary atom site location: difference Fourier map
*R*[*F*^2^ > 2σ(*F*^2^)] = 0.049	Hydrogen site location: inferred from neighbouring sites
*wR*(*F*^2^) = 0.134	H-atom parameters constrained
*S* = 1.02	*w* = 1/[σ^2^(*F*_o_^2^) + (0.0608*P*)^2^ + 0.7105*P*] where *P* = (*F*_o_^2^ + 2*F*_c_^2^)/3
1987 reflections	(Δ/σ)_max_ < 0.001
130 parameters	Δρ_max_ = 0.30 e Å^−^^3^
0 restraints	Δρ_min_ = −0.28 e Å^−^^3^

### Special details

**Table:**

Geometry. All e.s.d.'s (except the e.s.d. in the dihedral angle between two l.s. planes) are estimated using the full covariance matrix. The cell e.s.d.'s are taken into account individually in the estimation of e.s.d.'s in distances, angles and torsion angles; correlations between e.s.d.'s in cell parameters are only used when they are defined by crystal symmetry. An approximate (isotropic) treatment of cell e.s.d.'s is used for estimating e.s.d.'s involving l.s. planes.
Refinement. Refinement of *F*^2^ against ALL reflections. The weighted *R*-factor *wR* and goodness of fit *S* are based on *F*^2^, conventional *R*-factors *R* are based on *F*, with *F* set to zero for negative *F*^2^. The threshold expression of *F*^2^ > σ(*F*^2^) is used only for calculating *R*-factors(gt) *etc*. and is not relevant to the choice of reflections for refinement. *R*-factors based on *F*^2^ are statistically about twice as large as those based on *F*, and *R*- factors based on ALL data will be even larger.

### Fractional atomic coordinates and isotropic or equivalent isotropic displacement parameters (Å^2^)

**Table:**

	*x*	*y*	*z*	*U*_iso_*/*U*_eq_	
N1	0.4226 (3)	0.73560 (9)	0.54034 (9)	0.0197 (3)	
O1	0.7485 (3)	0.96228 (7)	0.64708 (8)	0.0207 (3)	
O2	0.5468 (3)	0.95300 (8)	0.78490 (8)	0.0255 (3)	
O3	−0.0290 (3)	0.75164 (8)	0.84444 (8)	0.0215 (3)	
H3	−0.0566	0.7606	0.8997	0.032*	
C1	0.5219 (4)	0.81023 (11)	0.58079 (11)	0.0184 (4)	
H1	0.6416	0.8453	0.5474	0.022*	
C2	0.4576 (3)	0.83885 (10)	0.66979 (11)	0.0167 (3)	
C3	0.2720 (3)	0.78652 (11)	0.72042 (10)	0.0167 (3)	
C4	0.1707 (4)	0.70920 (11)	0.67810 (11)	0.0189 (4)	
H4	0.0480	0.6732	0.7092	0.023*	
C5	0.2507 (4)	0.68475 (11)	0.58930 (11)	0.0191 (4)	
C6	0.1465 (5)	0.60006 (12)	0.54492 (12)	0.0290 (4)	
H6A	−0.0682	0.5987	0.5366	0.044*	
H6B	0.2204	0.5532	0.5854	0.044*	
H6C	0.2206	0.5942	0.4844	0.044*	
C7	0.1868 (4)	0.81091 (11)	0.81720 (11)	0.0184 (4)	
H7A	0.3629	0.8099	0.8633	0.022*	
H7B	0.1053	0.8691	0.8152	0.022*	
C8	0.5846 (3)	0.92273 (11)	0.70790 (11)	0.0184 (4)	
C9	0.8760 (4)	1.04629 (11)	0.67761 (12)	0.0218 (4)	
H9A	1.0137	1.0397	0.7348	0.026*	
H9B	0.7187	1.0860	0.6906	0.026*	
C10	1.0378 (4)	1.08024 (12)	0.59824 (12)	0.0242 (4)	
H10A	1.1936	1.0406	0.5864	0.036*	
H10B	1.1235	1.1357	0.6156	0.036*	
H10C	0.8994	1.0861	0.5421	0.036*	

### Atomic displacement parameters (Å^2^)

**Table:**

	*U*^11^	*U*^22^	*U*^33^	*U*^12^	*U*^13^	*U*^23^
N1	0.0253 (7)	0.0210 (7)	0.0131 (6)	0.0006 (5)	0.0027 (5)	0.0001 (5)
O1	0.0250 (6)	0.0200 (6)	0.0180 (6)	−0.0041 (5)	0.0057 (5)	−0.0031 (5)
O2	0.0332 (7)	0.0261 (7)	0.0180 (6)	−0.0048 (5)	0.0073 (5)	−0.0061 (5)
O3	0.0263 (6)	0.0275 (6)	0.0115 (5)	−0.0033 (5)	0.0060 (5)	−0.0002 (5)
C1	0.0216 (8)	0.0205 (8)	0.0137 (7)	−0.0002 (6)	0.0043 (6)	0.0020 (6)
C2	0.0169 (7)	0.0200 (8)	0.0127 (7)	0.0034 (6)	0.0001 (6)	0.0000 (6)
C3	0.0174 (7)	0.0216 (8)	0.0108 (7)	0.0038 (6)	0.0005 (6)	0.0028 (6)
C4	0.0223 (8)	0.0220 (8)	0.0123 (7)	−0.0013 (6)	0.0022 (6)	0.0032 (6)
C5	0.0237 (8)	0.0206 (8)	0.0127 (7)	0.0007 (6)	0.0005 (6)	0.0001 (6)
C6	0.0450 (11)	0.0257 (9)	0.0170 (8)	−0.0089 (8)	0.0062 (7)	−0.0030 (7)
C7	0.0215 (8)	0.0218 (8)	0.0123 (7)	−0.0003 (6)	0.0032 (6)	0.0003 (6)
C8	0.0191 (7)	0.0210 (8)	0.0151 (7)	0.0024 (6)	0.0021 (6)	0.0006 (6)
C9	0.0255 (8)	0.0184 (8)	0.0217 (8)	−0.0018 (6)	0.0026 (7)	−0.0035 (6)
C10	0.0270 (8)	0.0234 (9)	0.0219 (8)	−0.0051 (7)	0.0015 (7)	−0.0014 (7)

### Geometric parameters (Å, °)

**Table:**

N1—C1	1.342 (2)	C4—H4	0.9300
N1—C5	1.349 (2)	C5—C6	1.504 (2)
O1—C8	1.344 (2)	C6—H6A	0.9600
O1—C9	1.4649 (19)	C6—H6B	0.9600
O2—C8	1.219 (2)	C6—H6C	0.9600
O3—C7	1.420 (2)	C7—H7A	0.9700
O3—H3	0.8200	C7—H7B	0.9700
C1—C2	1.400 (2)	C9—C10	1.503 (2)
C1—H1	0.9300	C9—H9A	0.9700
C2—C3	1.415 (2)	C9—H9B	0.9700
C2—C8	1.493 (2)	C10—H10A	0.9600
C3—C4	1.391 (2)	C10—H10B	0.9600
C3—C7	1.515 (2)	C10—H10C	0.9600
C4—C5	1.402 (2)		
			
C1—N1—C5	117.54 (14)	H6B—C6—H6C	109.5
C8—O1—C9	115.95 (13)	O3—C7—C3	109.70 (13)
C7—O3—H3	109.5	O3—C7—H7A	109.7
N1—C1—C2	124.43 (15)	C3—C7—H7A	109.7
N1—C1—H1	117.8	O3—C7—H7B	109.7
C2—C1—H1	117.8	C3—C7—H7B	109.7
C1—C2—C3	118.16 (15)	H7A—C7—H7B	108.2
C1—C2—C8	119.48 (14)	O2—C8—O1	122.94 (15)
C3—C2—C8	122.36 (14)	O2—C8—C2	124.91 (15)
C4—C3—C2	117.04 (14)	O1—C8—C2	112.14 (13)
C4—C3—C7	120.10 (14)	O1—C9—C10	107.08 (13)
C2—C3—C7	122.86 (14)	O1—C9—H9A	110.3
C3—C4—C5	121.03 (15)	C10—C9—H9A	110.3
C3—C4—H4	119.5	O1—C9—H9B	110.3
C5—C4—H4	119.5	C10—C9—H9B	110.3
N1—C5—C4	121.78 (15)	H9A—C9—H9B	108.6
N1—C5—C6	117.43 (14)	C9—C10—H10A	109.5
C4—C5—C6	120.79 (15)	C9—C10—H10B	109.5
C5—C6—H6A	109.5	H10A—C10—H10B	109.5
C5—C6—H6B	109.5	C9—C10—H10C	109.5
H6A—C6—H6B	109.5	H10A—C10—H10C	109.5
C5—C6—H6C	109.5	H10B—C10—H10C	109.5
H6A—C6—H6C	109.5		
			
C5—N1—C1—C2	−0.4 (2)	C3—C4—C5—N1	−1.5 (2)
N1—C1—C2—C3	−0.9 (2)	C3—C4—C5—C6	178.53 (15)
N1—C1—C2—C8	179.21 (14)	C4—C3—C7—O3	−8.1 (2)
C1—C2—C3—C4	1.0 (2)	C2—C3—C7—O3	172.83 (13)
C8—C2—C3—C4	−179.12 (14)	C9—O1—C8—O2	−1.5 (2)
C1—C2—C3—C7	−179.91 (14)	C9—O1—C8—C2	178.51 (12)
C8—C2—C3—C7	0.0 (2)	C1—C2—C8—O2	−178.81 (16)
C2—C3—C4—C5	0.1 (2)	C3—C2—C8—O2	1.3 (2)
C7—C3—C4—C5	−178.98 (14)	C1—C2—C8—O1	1.2 (2)
C1—N1—C5—C4	1.6 (2)	C3—C2—C8—O1	−178.66 (13)
C1—N1—C5—C6	−178.42 (15)	C8—O1—C9—C10	−177.70 (13)

### Hydrogen-bond geometry (Å, °)

**Table:**

*D*—H···*A*	*D*—H	H···*A*	*D*···*A*	*D*—H···*A*
O3—H3···N1^i^	0.82	2.01	2.8227 (17)	170.

Symmetry codes: (i) *x*−1/2, −*y*+3/2, *z*+1/2.
